# Lateralization and Bodily Patterns of Segmental Signs and Spontaneous Pain in Acute Visceral Disease: Observational Study

**DOI:** 10.2196/27247

**Published:** 2021-08-27

**Authors:** Nour Shaballout, Anas Aloumar, Jorge Manuel, Marcus May, Florian Beissner

**Affiliations:** 1 Somatosensory and Autonomic Therapy Research Institute for Neuroradiology Hannover Medical School Hannover Germany; 2 Department of Internal Medicine Klinikum Region Hannover Großburgwedel Hannover Germany; 3 Institute of Aerospace Medicine German Aerospace Centre Cologne Germany; 4 Insula Institute for Integrative Therapy Research Hannover Germany

**Keywords:** digital pain drawings, visceral referred pain, referred pain, head zones, mydriasis, chest pain, clinical examination, differential diagnosis, digital health, digital drawings, pain, health technology, image analysis

## Abstract

**Background:**

The differential diagnosis of acute visceral diseases is a challenging clinical problem. Older literature suggests that patients with acute visceral problems show segmental signs such as hyperalgesia, skin resistance, or muscular defense as manifestations of referred visceral pain in somatic or visceral tissues with overlapping segmental innervation. According to these sources, the lateralization and segmental distribution of such signs may be used for differential diagnosis. Segmental signs and symptoms may be accompanied by spontaneous (visceral) pain, which, however, shows a nonsegmental distribution.

**Objective:**

This study aimed to investigate the lateralization (ie, localization on one side of the body, in preference to the other) and segmental distribution (ie, surface ratio of the affected segments) of spontaneous pain and (referred) segmental signs in acute visceral diseases using digital pain drawing technology.

**Methods:**

We recruited 208 emergency room patients that were presenting for acute medical problems considered by triage as related to internal organ disease. All patients underwent a structured 10-minute bodily examination to test for various segmental signs and spontaneous visceral pain. They were further asked their segmental symptoms such as nausea, meteorism, and urinary retention. We collected spontaneous pain and segmental signs as digital drawings and segmental symptoms as binary values on a tablet PC. After the final diagnosis, patients were divided into groups according to the organ affected. Using statistical image analysis, we calculated mean distributions of pain and segmental signs for the heart, lungs, stomach, liver/gallbladder, and kidneys/ureters, analyzing the segmental distribution of these signs and the lateralization.

**Results:**

Of the 208 recruited patients, 110 (52.9%) were later diagnosed with a single-organ problem. These recruited patients had a mean age of 57.3 (SD 17.2) years, and 40.9% (85/208) were female. Of these 110 patients, 85 (77.3%) reported spontaneous visceral pain. Of the 110, 81 (73.6%) had at least 1 segmental sign, and the most frequent signs were hyperalgesia (46/81, 57%), and muscle resistance (39/81, 48%). While pain was distributed along the body midline, segmental signs for the heart, stomach, and liver/gallbladder appeared mostly ipsilateral to the affected organ. An unexpectedly high number of patients (37/110, 33.6%) further showed ipsilateral mydriasis.

**Conclusions:**

This study underlines the usefulness of including digitally recorded segmental signs in bodily examinations of patients with acute medical problems.

## Introduction

The differential diagnosis of acute visceral diseases is a common but challenging clinical problem. Since pain originating from visceral organs (ie, visceral pain) often exhibits characteristic patterns [1-8], many textbooks assign pain location a discriminative role in the differential diagnosis [9,10]. However, many studies have also reported negative results when testing the predictive power of pain location [11,12]. For example, pain localization in patients with coronary heart disease does not significantly differ from chest pain patients without coronary heart disease [13].

While primary visceral pain itself is a poorly defined, midline sensation, it starts to be referred or “transferred” to somatic structures when it persists for several minutes or longer [14,15]. These somatic structures can include skin, subcutaneous tissue, and muscle and are characterized by an overlapping segmental innervation with that of the diseased organ [16-29]. In these instances, referred visceral pain manifests as hyperalgesia, a phenomenon first described by Ross and Sturge in the 1880s [30,31] and subsequently studied in depth by Head and Mackenzie [16-19]. Head mapped out the cutaneous zones of referred hyperalgesia for all major organs and compared them with the location of skin lesions in herpes zoster [16]. The result is still considered one of the most precise maps of segmental innervation [32,33].

To the present day, zones of referred hyperalgesia in visceral disease carry Head’s name in many European countries, such as France, Germany, and Spain. In other parts of the world, however, clinicians mainly speak of “dermatomes,” and clinicians hardly know the term “Head zones” as well as Head’s work, in general. Some authors have even called segmental anatomy a “wrongly forgotten science” [29]. Only rarely do clinicians know that the transmitted signs are not limited to hyperalgesia of the skin but instead show a plethora of manifestations, including sensory disturbances such as allodynia and deep hyperalgesia (ie, Mackenzie zones); motor disturbances such as increased resistance of the skin, muscular defense, and resistance to passive joint movement; and, finally, signs of sympathetic activation such as vasomotor changes, asymmetric hyperhidrosis (ie, asymmetric sweating between left and right side of body), piloerection, and anisocoria (ie, unequal pupil size). As such, they are not limited to the dermatomes but include the myotomes, sclerotomes, and other parts of the segmental innervation [4]. Furthermore, segmental signs may be accompanied by symptoms of viscero-visceral reflexes such as nausea, vomiting, diarrhea, constipation, meteorism, and urinary retention [33].

To our knowledge, a systematic evaluation of simultaneously collected segmental signs and symptoms in patients has never been published in the English scientific literature. In Germany, however, Karl Hansen (1893-1962) and Hans Schliack (1919-2008) had studied a wide variety of segmental signs over several decades. While their results have only been published in German [33], the essence of their work has recently been made available in book form and extended by the work of other clinicians [29]. In a large sample of internal medicine patients, Hansen and Schliack [33] confirmed many of Head’s observations and greatly extended them to include all of the above-mentioned segmental signs and symptoms. Even more than Head, the authors emphasized the importance of sign lateralization by defining a side rule, according to which segmental signs are most likely to appear ipsilateral to the affected organ (Table 1).

**Table 1:** Lateralization of segmental signs for individual organs according to Hansen and Schliack [33].

Organ	Segmental signs by side of body and part of organ (yes, possible,^a^ or no^b^)	
	Right	Left
Heart	Possible	Yes
Pericardium	No	Yes
Aorta	Possible	Yes
Lung and bronchi	Yes	Yes
Pleura	Yes	Yes
Stomach	Yes (pylorus)	Yes (corpus, fundus)
Small intestine	Yes (duodenum, ileum)	Yes (jejunum)
Pancreas	No	Yes
Liver	Yes	No
Gallbladder	Yes	No
Spleen	No	Yes
Large intestine	Yes (caecum, appendix, ascending colon, proximal part of transverse colon)	Yes (distal part of transverse colon, descending colon, sigmoid colon, rectum)
Kidney	Yes	Yes
Ureter	Yes	Yes
Testis and ovary	Yes (testis, ovary, salpinx)	Yes (testis, ovary, salpinx)

^a^Possible indicates a possible but unlikely occurrence of signs from that organ.

^b^No indicates that segmental signs from a particular organ were never observed on that side.

A methodological problem that has hampered clinical research of segmental signs for a long time is the difficulty in adequately measuring bodily signs. However, recent developments in the field of digital pain drawings offer new and exciting possibilities to systematically record not only pain sensations but also segmental signs and analyze them using methods of statistical image analysis [34].

Here, we report the results of a study that investigated both the bodily patterns and lateralization of segmental signs and spontaneous pain in acute visceral diseases. We aimed to derive mean distributions of spontaneous pain and segmental signs for as many internal organs as possible and to analyze their segmental content and lateralization. To achieve this, we combined digital pain drawing technology and a structured, 10-minute bodily examination in a sample of emergency room patients.

## Methods

### Ethics

The study was approved by the Ethics Committee of Hannover Medical School (number 2987-2017) and was conducted under the Declaration of Helsinki. All patients provided written informed consent after they were informed about the purpose of the study.

### Study Population

**Figure 1:**
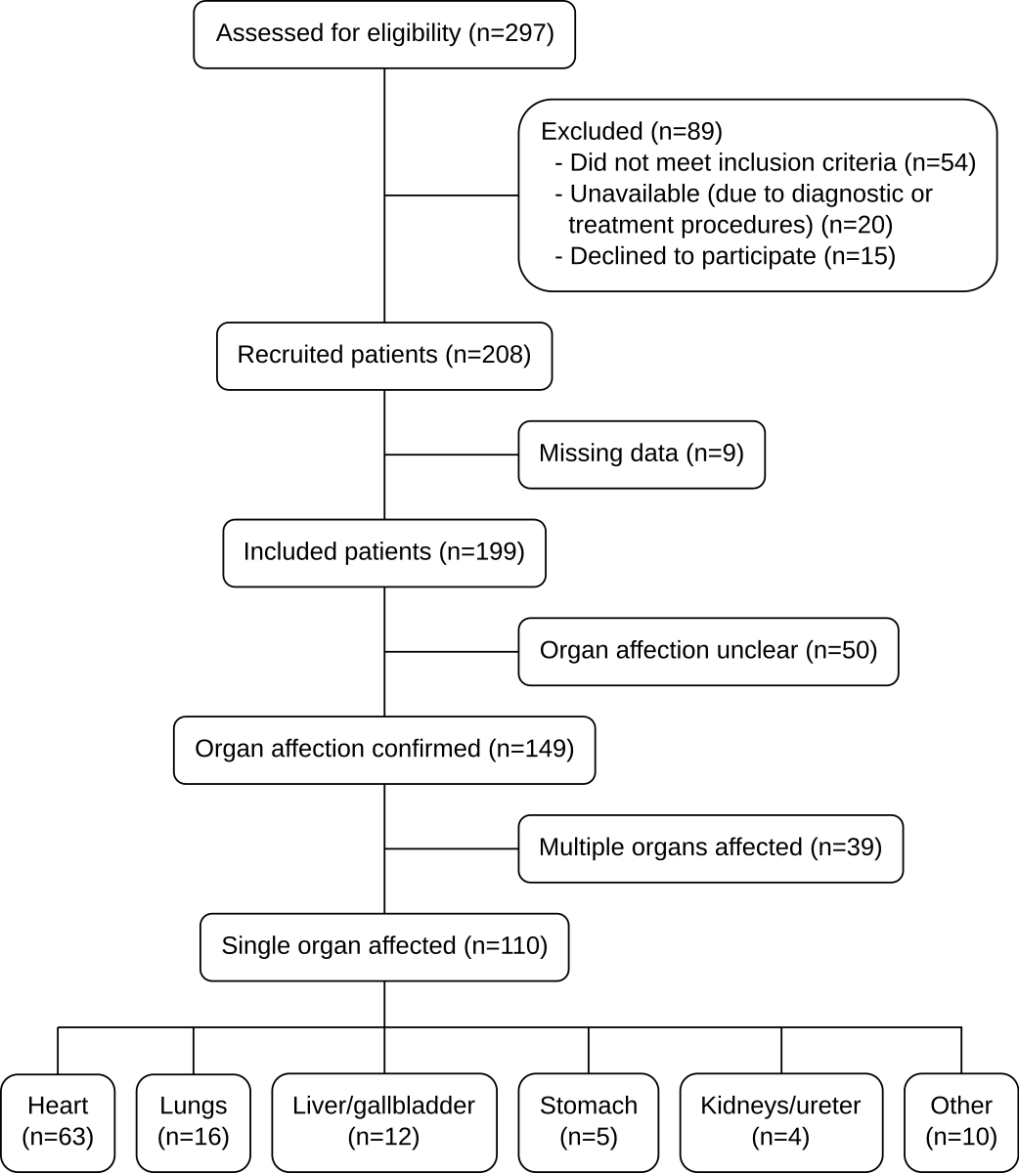
Flowchart of the study.

Our study population consisted of patients from the emergency department of Hannover Medical School who were referred to internal medicine physicians between March 2017 and October 2017. Eligible patients were adults (age ≥18 years in Germany), presenting with an acute medical problem and with the ability to provide written informed consent. Furthermore, patients needed to be oriented as to place, time, and person. Exclusion criteria comprised refusal or inability to provide written consent, previously known or acutely diagnosed spinal cord injury, pregnancy, acute or past ocular illnesses, acute or past central or peripheral nervous disease, uncooperative patients, and patients who only presented to the emergency room for educational purposes or to receive a prescription. For a flowchart, see Figure 1.

We recruited 208 patients (85, 40.9% women) for participation in our study. Nine drawings were lost due to technical failure of a tablet PC during the physical examination. The characteristics of the final study population can be found in Table 2, and their final diagnoses can be found in Multimedia Appendix 1.

**Table 2:** Demographics of the study population (n=208).

Characteristic		Value
Age (years), mean (SD)		57.3 (17.2)
**Age range, n (%)**		
	18-39	34 (17.1)
	40-59	66 (33.2)
	60-79	80 (40.2)
	≥80	19 (9.5)
**Gender, n (%)**		
	Women	85 (40.9)
	Men	123 (59.1)
**Main complaint, n (%)**		
	Chest pain	88 (44.2)
	Abdominal pain	55 (27.6)
	Dyspnea	22 (11.1)
	Other	34 (17.1)

### Procedures

All clinical data were collected by 2 of the authors (AA and NS), henceforth called examiners. AA is an internal medicine specialist, and NS is a physician with 4 years of training for an internal medicine specialization. The examiners were fully informed about the study purpose and trained to do the physical examination for segmental signs and symptoms according to the protocol described below. Prior to the study, the examiners trained intensively together to ensure their physical examinations were standardized. This was also necessary to ensure that all procedures could be completed in a very limited timeframe.

During recruitment, the examiners screened the emergency dashboard to identify patients who were referred to internal medicine specialists. They approached all eligible patients, informed them about the study, and obtained written informed consent.

The examination took place directly after triage and before any medical intervention, diagnosis, or treatment. The examination lasted between 7 and 15 minutes, depending on the patient’s compliance (ie, general motivation to be examined, speed of undressing and answering the examiner’s questions, precision of the answers given, unrelated conversation, etc) and interruptions by nurses and physicians (as routine diagnostics and medical interventions had priority over the scientific investigation). Directly after the physical examination, all findings were recorded on a tablet computer running the app “SymptomMapper” (described in the section “Tablet Computer and Software Application”).

### Categories of Findings

The clinical findings we were interested in can be divided into 3 groups, according to the ways they were recorded in the tablet: (1) distributed findings (ie, those with a bodily pattern), (2) lateralized findings (ie, those without a bodily pattern but with clear lateralization), and (3) other findings.

First, distributed findings were spontaneous pain, allodynia, superficial hyperalgesia, deep hyperalgesia, superficial skin resistance, muscle resistance, defense, asymmetric hyperhidrosis, piloerection, vasomotor changes, herpes zoster, and resistance to passive movement of the limbs. Distributed findings were recorded by the examiners in the form of electronic drawings on a body template, thus capturing their exact location and extent.

Second, lateralized findings were anisocoria, glossy eye, eyelid separation, tense facial muscles, asymmetric posture, and reduced respiration movements. These findings were recorded by choosing from a list of the abovementioned findings in conjunction with a side label (eg, “glossy eye right,” “mydriasis left,” etc).

Third, other findings were symptoms potentially related to viscero-visceral reflexes, namely, nausea, vomiting, constipation, diarrhea, meteorism, and urinary retention. These findings were recorded by choosing from a simple list of the abovementioned symptoms.

### Tablet Computer and Software Application

All findings were recorded on Galaxy Note 10.1 (2014) tablet PCs with an electronic stylus based on inductive digitizing technology (Samsung, Seoul, South Korea). The tablets had a 10.1-inch touch screen with a resolution of 800×1280 pixels and were running Android 5.1.1 (Open Handset Alliance, Mountain View, CA, United States). The stylus was used for all data entry, hence allowing for a higher resolution while eliminating unwanted activation of the screen, for example, by the palm. The tablet and stylus were disinfected after every patient using disinfectant wipes.

We used a modified version of the SymptomMapper app developed by our group (Somatosensory and Autonomic Therapy Research, Institute for Neuroradiology, Hannover Medical School, Hannover, Germany) to acquire electronic pain drawings [35]. Its usability for doctors and the reliability of its symptom-drawing approach have recently been shown [35]. The app allowed the examiners to enter all findings from the bodily examination quickly. They could either draw distributed findings on a body template or choose from a list of lateralized or other findings. For the electronic drawings, examiners had a front and back view of the body available, and each newly added sign or symptom was displayed in a semitransparent way and in a different color.

### Bodily Examination

#### Distributed Segmental Sign Examination

Our approach to the bodily examination was based entirely on Hansen and Schliack (p140-176) [33]. Its primary purpose was to check for the presence and record the extent and lateralization of pain and segmental signs and symptoms. We start by describing how distributed segmental signs were collected, as this was the same for different body regions (see below). These collection methods were (1) visual inspection and (2) palpation.

First, for visual inspection, the skin was visually inspected for the following signs: shingles (as a potential sign of Zoster reactivation), vasomotor changes (ie, skin color changing to red, pale, or blue, as a sign of sympathetic reflexes); piloerection (ie, any hair erection or “goosebumps,” as a sign of sympathetic reflexes), and muscular asymmetries (eg, asymmetric posture, tense facial muscles, respiratory chest movement, etc).

Second, for palpation, the body was palpated with warm hands to test for the following signs of sympathetic reflexes, increased muscle tone, or sensory disturbance: (1) asymmetric hyperhidrosis, (2) superficial hyperalgesia, (3) deep hyperalgesia, (4) allodynia, (5) superficial skin resistance, and (6) muscle resistance. Among palpation, first, for asymmetric hyperhidrosis, the skin was observed and palpated for any local differences in the amount of sweating. Second, for superficial hyperalgesia, the patient was informed that the examination could cause a little twinge and then was asked if the tip of a neurological examination needle (Healthstar, Lakewood, NJ, United States), when passed vertically over the skin in long and slow strokes, caused a different sensation in any area. Third, for deep hyperalgesia, folds of skin were held gently between the thumb and index finger or the region was tapped on. The test was considered positive if this procedure caused dull pain that lasted longer in some part of the body than in other parts of the body. Fourth, for allodynia, patients were asked if their clothes caused an unpleasant sensation somewhere on the body. Then, they were asked if a medical cotton swab passed over the skin in long and slow strokes caused a different sensation in any area. Fifth, superficial skin resistance was tested by superficial palpation of the trunk skin using the palm with very soft pressure. If the examiner felt either resistance or a rubbery membrane in any area, the test was considered positive in this area. Sixth, for muscle resistance, deep palpation of the trunk wall was performed on the front and back sides with the palm to detect the guarding of the trunk’s wall muscles (ie, anterior thoracic muscles, anterolateral abdominal wall muscles, posterior superficial muscles, and posterior deep muscles).

#### Complete Examination

The complete examination program had the following steps carried out in the exact order specified here: examination of (1) asymmetric posture, (2) pain and segmental symptoms, (3) the head, (4) the neck and chest, (5) the abdomen, and (6) the limbs.

First, for asymmetric posture, a general visual inspection of the patient’s posture was carried out directly after entering the examination room to check side differences in muscle tone.

Second, pain and segmental symptoms were collected by asking patients the following questions: (1) “Do you have pain—where, exactly? Do you have a headache?” and, when the patient reported pain, the painful region was drawn; (2) “Do you have nausea? Did you vomit since the onset of symptoms?”; (3) “Do you have diarrhea or constipation?”; (4) “Do you feel that your abdomen is full of gas?”; and (4) “Did you have any problem with urination since the onset of symptoms?”

Third, the head was examined with (1) special tests and (2) tests of distributed segmental signs. For the head, first, the special tests examined (1) the pupils, (2) the eyes/eyelids, and (3) tense facial muscles. For these special tests, first, for pupils, we tested for mydriasis, a sign of sympathetic activation, by equally exposing both eyes to light after instructing the patient to relax and look far away. The examiner used one hand to shadow the eyes and compared pupil diameters on both sides. This was repeated 3-5 times. In the case of a striking side difference, the test was considered positive for mydriasis. Second, for eyes/eyelids, eyelid separation (due to eyelid retraction) and eye gloss (due to excessive lacrimation) are both signs of sympathetic activation and were assessed by visually comparing the visible area and gloss of both eyes. In the case of a striking side difference, the more open and glossier eye was noted. Third, for tense facial muscles, a potential asymmetry of facial features caused by side differences in muscle tone was checked visually. It was considered positive when the upper lip was noticeably higher, the nasolabial fold deeper, and the cheeks more retracted on one side than on the other. The test was repeated once under provocation by applying pressure with the index and middle fingers on a point between the 2 heads of the sternocleidomastoid muscle. In terms of head tests overall, second, the distributed segmental signs were tested, including zoster, vasomotor changes, piloerection, asymmetric hyperhidrosis, superficial hyperalgesia, allodynia, and superficial skin resistance.

Fourth, as part the complete examination, was the neck and chest, where the patient’s front was examined while the patient was in a supine position after freeing the chest from clothes. Then, the back was examined with the patient sitting or lying on one side. Similar as for the head, the neck and chest included special tests (ie, the patient's chest movement during inspiration and expiration was observed during the visual inspection over several respiratory cycles, and any striking side differences were noted as a sign of increased muscle tone) and distributed segmental sign tests (ie, tests for zoster, vasomotor changes, piloerection, asymmetric hyperhidrosis, superficial hyperalgesia, deep hyperalgesia, allodynia, superficial skin resistance, and muscle resistance).

Fifth, for the abdomen, the patient was examined on the front in a supine position after freeing the abdomen. Then, the back was examined with the patient sitting or lying on one side. Again, special tests were performed; mainly, the defense was examined by applying sudden deep palpation over the painful areas of the abdomen. If the examiner felt a reflex of the abdominal wall, it was considered positive. Next, distributed segmental signs were tested: zoster, vasomotor changes, piloerection, asymmetric hyperhidrosis, superficial hyperalgesia, deep hyperalgesia, allodynia, superficial skin resistance, and muscle resistance.

Sixth, for limbs, as for the other body parts, there were special tests and testing of distributed segmental signs. For special tests, passive movements of the joints were examined to detect any resistance due to increased muscle tone. The distributed segmental signs test included zoster, vasomotor changes, piloerection, superficial hyperalgesia, and allodynia.

### Patient Selection

Medical reports of all recruited patients were followed up through Hannover Medical Schools’ electronic health records by 3 of the authors (NS, AA, and MM), to identify those patients with a definite diagnosis of visceral disease. All information regarding the acute complaint, previous diagnoses, and diagnostic procedures (electrocardiogram, laboratory, radiology, etc) were reviewed, and the most likely etiology for each patient was discussed. Patients without a definite visceral diagnosis were excluded from further analysis (n=50). The remaining cases were divided into those where a single organ was affected (n=110) and those with multi-organ problems (n=39). Only the single-organ cases were included in the final analysis, and only organs with at least 4 patients in the sample were included in any organ-specific analyses (Figure 1).

### Data Analysis

#### General Considerations on Lateralization

According to Hansen and Schliack [33], the majority of segmental signs are lateralized and appear on specific sides of the body defined by the innervation of the individual organs (Table 1). In particular, the lateralization of signs for paired organs such as lungs and kidneys depends on which side is affected. Due to the nature of this study, it was not possible to conduct separate analyses for the left and right side in diseases of the lungs and kidneys/ureters. Furthermore, many lung cases were bilateral affections. Information about the lateralization of segmental signs for lungs and kidneys/ureters is, therefore, of little value and only shown for the sake of completeness.

#### Lateralization and Other Findings

We extracted all lateralized findings (ie mydriasis, glossy eye, eyelid separation, tense facial muscles, asymmetric posture, and reduced respiration movements) and other findings (nausea, vomiting, constipation, diarrhea, meteorism, and urinary retention) from SymptomMapper’s JavaScript Object Notation files using a custom-written Python script (Python 2.7, Python Software Foundation, 2018). Then, we calculated, for each organ and each finding, the percentage of patients that had that finding. For lateralized findings, we calculated the percentage for each side individually, treating front and back as one surface. Finally, we calculated the mean frequency of each finding (ie, how often it was observed), irrespective of the specific organ.

#### Distributed Findings

Digital drawings from the app were converted to Nifti format (Neuroimaging Informatics Technology Initiative, 2017) with a custom-written Python script (Python 2.7, Python Software Foundation, 2018) and analyzed using tools from the Functional Magnetic Resonance Imaging (FMRIB) Software Library (FSL) version 5.0 (FMRIB Analysis Group, Oxford University, United Kingdom). Figures were prepared using VINCI (Volume Imaging in Neurological Research, Co-Registration and Region of Interest (ROI)s Included) 4.86.0 (Max Planck Institute for Metabolism Research, Cologne, Germany) and GNU Image Manipulation Program (GIMP; version 2.8.16, The GIMP Team).

First, to derive the bodily distribution of all segmental signs, all distributed signs were superimposed and the result binarized. In the resulting map, a pixel of value 1 on the body template meant that at least 1 sign had been found at that particular point on the body, in that particular patient. Binarization meant that we disregarded the number of signs that each patient showed and instead only considered their bodily location.

We then analyzed distributed signs individually to assess the segmental distribution for each sign according to the segmental scheme of Hansen and Schliack [33], which is largely based on Head’s scheme [14,32]. To do this assessment, we calculated, for each segment, the percentage of the segment covered by the sign. For this calculation, we divided the pixel count by the total number of pixels of the respective segment. Only segments with at least 5% coverage were included. This arbitrary threshold was set to ensure that segments with marginal coverage (eg, due to drawing imperfections) were excluded. To assess the lateralization of findings, we further divided segments into left and right body halves, calculating the percentage for each of them. This resulted in a list of half segments covered by each sign. Finally, we calculated, for each organ, the mean number of segmental signs per half segment and the mean frequency of each sign across all organs.

Spontaneous pain was analyzed in the same way but separately from all other signs.

## Results

### The Overall Frequency of Signs and Symptoms

Of the 110 patients in our final sample, 85 (77.3%) had spontaneous pain, 81 (73.6%) showed at least 1 segmental sign, and 52 (47.3%) showed at least 1 segmental symptom. On average, each patient had a mean of 1.80 (SD 1.86) segmental signs and 0.77 (SD 1.00) segmental symptoms. The most frequent signs and symptoms are shown in Table 3.

**Table 3:** Frequency of segmental signs and symptoms in our patient sample (N=110).

Segmental sign or symptom		Value, n (%)
**Segmental sign**		
	Superficial hyperalgesia (Head zone)	46 (41.8)
	Muscle resistance	39 (35.5)
	Mydriasis	37 (33.6)
	Defense	13 (11.8)
	Deep hyperalgesia (Mackenzie zone)	13 (11.8)
	Superficial skin resistance	12 (10.9)
	Tense facial muscles	11 (10.0)
	Vasomotor changes	10 (9.1)
	Glossy eye/wide eyelid	8 (7.3)
	Asymmetric posture	7 (6.4)
	Reduced respiration movements	3 (2.7)
	Allodynia	2 (1.8)
	Piloerection	1 (0.9)
	Asymmetric hyperhidrosis	1 (0.9)
	Zoster	0 (0)
At least 1 segmental sign		84 (76.4)
**Segmental symptoms**		
	Nausea	45 (40.9)
	Vomiting	18 (16.4)
	Diarrhea	10 (9.1)
	Meteorism	8 (7.3)
	Constipation	5 (4.5)
	Urinary retention	0 (0)
At least 1 segmental symptom		52 (47.3)
Spontaneous pain		85 (77.3)

### Frequency of Lateralization of Signs and Symptoms

All lateralization of signs and segmental symptoms are shown in Multimedia Appendix 2. As predicted by the side rule (see [33] and Table 1), the majority of lateralization signs were ipsilateral to the affected organ for the unpaired organs heart, stomach, and liver/gallbladder. The most striking finding was the high number of patients showing ipsilateral mydriasis as a potential sign of unilateral sympathetic activation. This lateralization was 100% ipsilateral for diseases of the liver/gallbladder (5 right vs 0 left), 100% ipsilateral for stomach diseases (1 left vs 0 right), and 83% ipsilateral for heart diseases (15 left vs 3 right).

### Segmental Signs and Spontaneous Pain in Individual Patients

**Figure 2:**
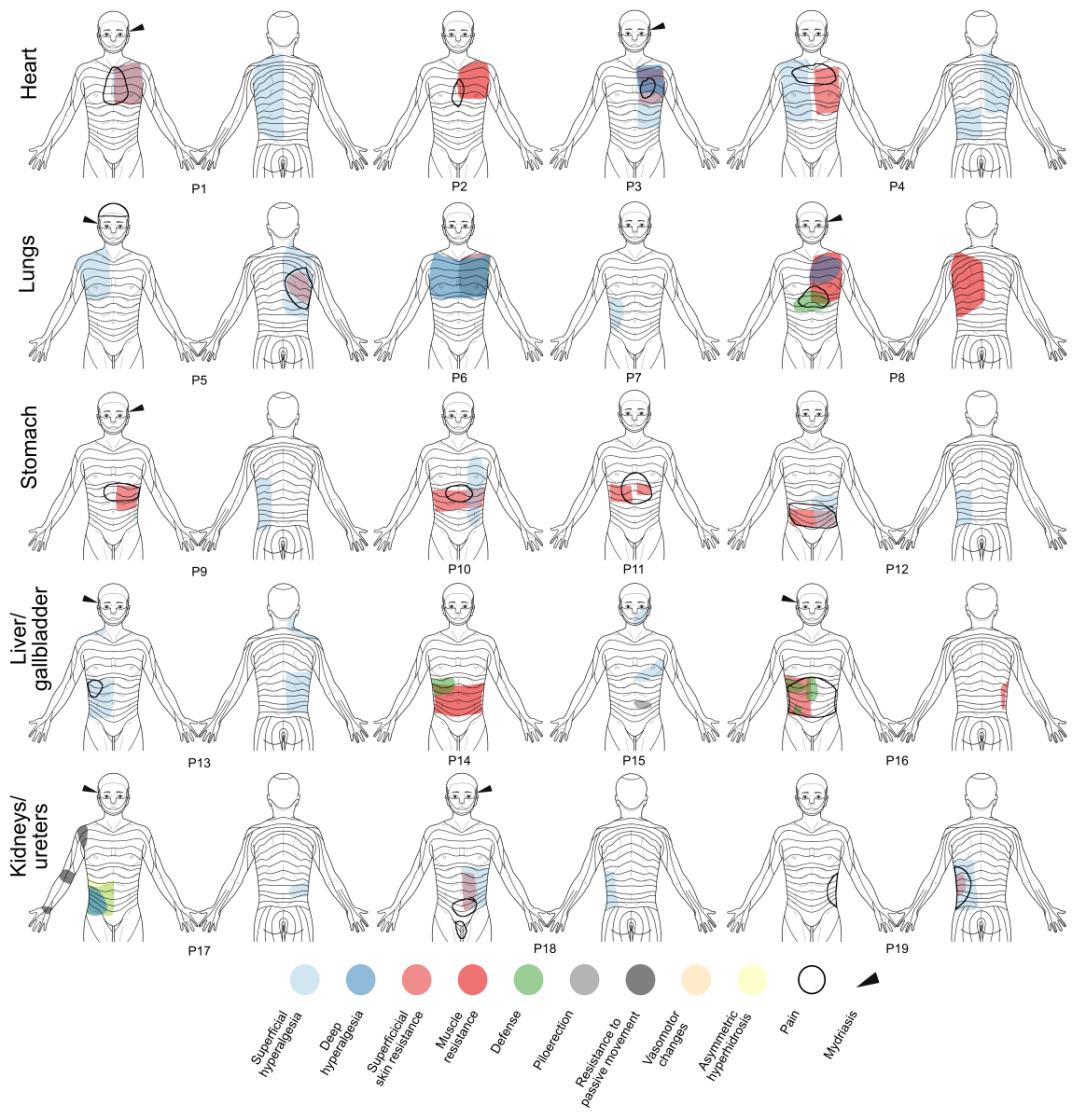
Segmental signs and spontaneous pain in individual patients with acute visceral diseases. P: patient.

Bodily maps of segmental signs and spontaneous pain for a representative selection of individual patients are shown in Figure 2. The cases shown in Figure 2 reflect the entire bandwidth of segmental signs encountered in patients presenting to the emergency room. It ranges from “textbook cases” (eg, patients 2, 3, 6, 9, 13, 18, and 19), where segmental signs alone allow for a preliminary diagnosis, to those where segmental signs are hardly helpful or even misleading (eg, patients 7, 12, and 15). Their primary diagnoses and demographic information are summarized in Multimedia Appendix 3.

### Bodily Maps and Segmental Patterns of Distributed Signs

**Figure 3:**
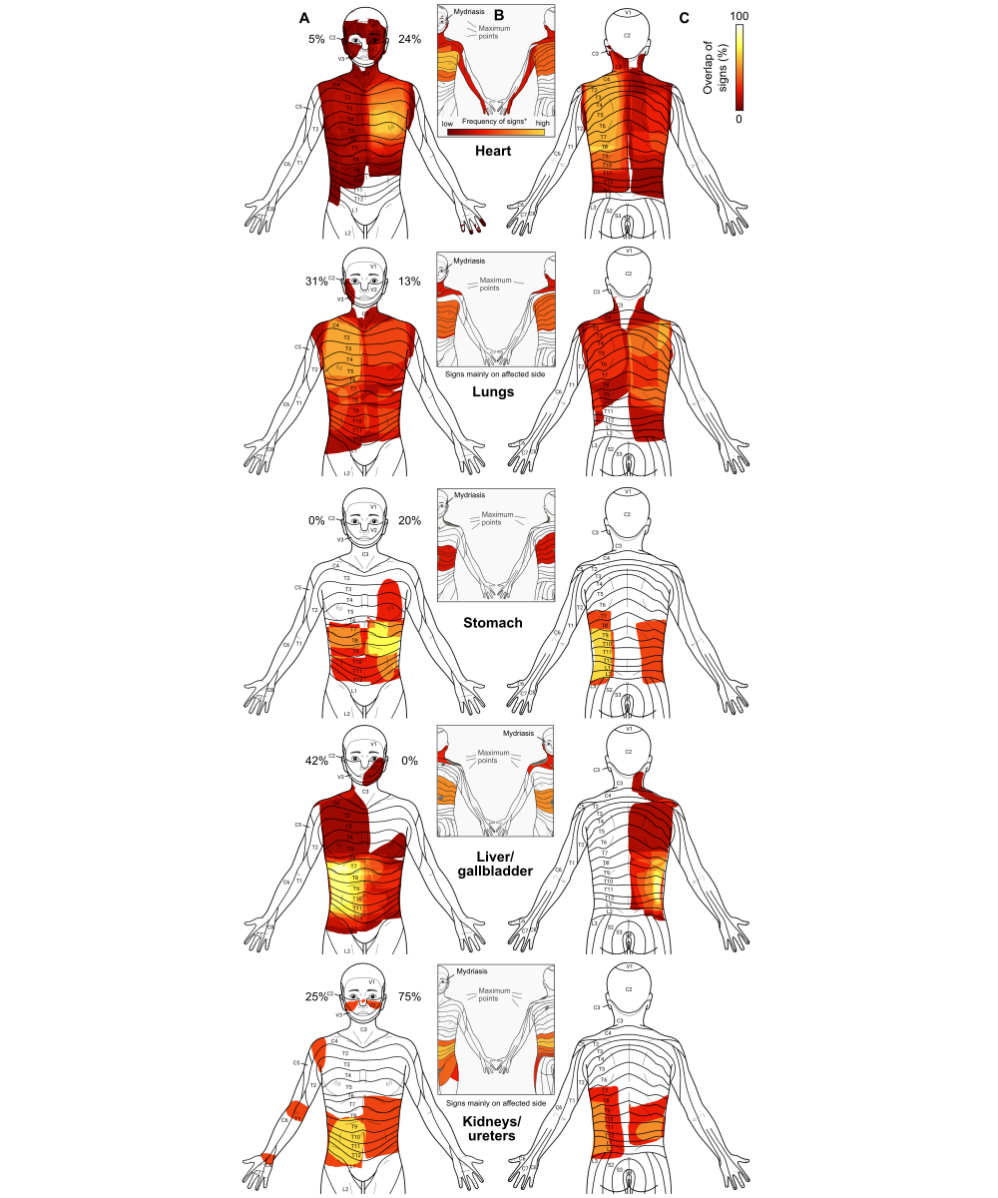
Distributed segmental signs in acute visceral diseases. Columns A and C show a front and back body map of all distributed segmental signs. The inserts in column B show the segmental distributions for each organ as reported by Hansen and Schliack [33], for comparison. Percentage values at the sides of the head indicate the frequency of unilateral mydriasis in affections of the respective organ.

**Figure 4:**
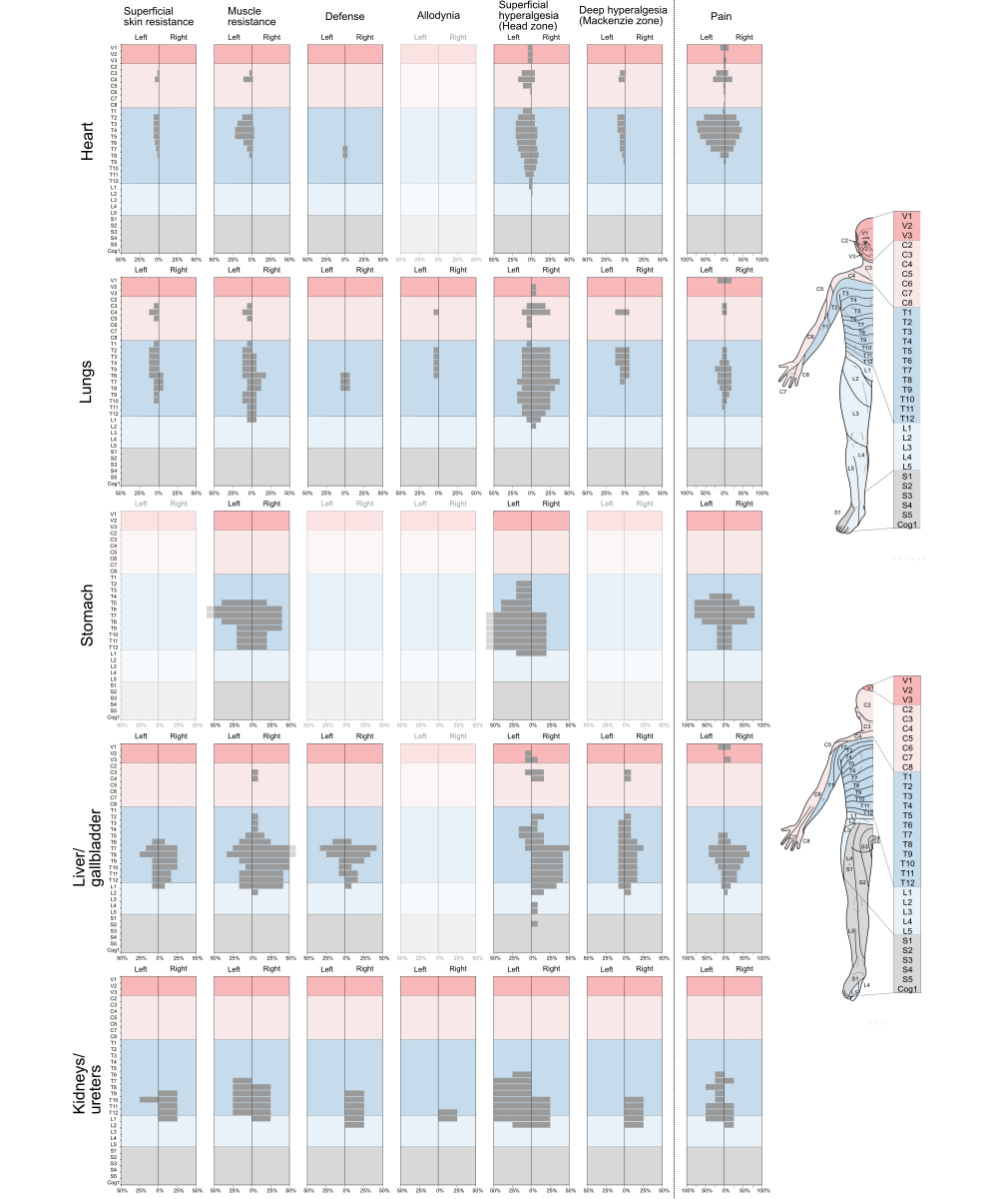
Segmental patterns and lateralization of segmental signs and pain in acute visceral diseases. Each row contains the results for an individual organ, while columns represent the different most-common segmental signs and pain, respectively. Graphs without any occurrence were blanked. Please note the different axis scaling for the latter. For the sake of clarity, segmental sections have been color-coded according to the body template shown on the right. This body template shows the different segments: trigeminal (V1-3), cervical (C2-8), thoracal (T1-12), lumbar (L1-5), sacral (S1-5), and coccygeal (Cog1).

Bodily maps of all distributed segmental signs are shown in Figure 3, while Figure 4 contains detailed segmental information concerning the distribution of the individual signs and spontaneous pain. In general, the observed distributions of segmental signs were largely consistent with those reported by Hansen and Schliack [33]. The lungs were the only exception, which showed a more widespread distribution than predicted. Concerning lateralization, segmental signs from the unpaired organs showed a clear side difference, with more signs appearing ipsilateral to the affected organ, thus supporting the “side rule” represented in Table 1. For the lungs and kidneys/ureters, however, this rule could not be tested, since results for these organs reflected a mixture of left, right, and bilateral affections.

Within-organ comparison showed that the different segmental signs but also spontaneous pain had a similar segmental distribution (Figure 4). Between organs, these distributions showed considerable overlap. Superficial hyperalgesia (Head zone) exhibited the greatest spread in terms of segments. Regarding lateralization, segmental signs for the unpaired organs mostly obeyed the side rule, according to which signs should appear on the body half where the organ is located. Pain differed markedly in that respect and, instead, showed a rather symmetric pattern.

For cardiac-related conditions, segmental signs were mostly located in the thoracic segments and, to a lesser extent, in cervical segments. Superficial hyperalgesia (Head zone) was also detected in the trigeminal segments. The maximum of the averaged signs was in the T3-T5 region (Figure 3B), as predicted by Hansen and Schliack [33]. In terms of lateralization, all distributed signs were strongly left-dominated with deep hyperalgesia (Mackenzie zone), showing complete left-lateralization (Figure 4). In heart patients, defense and allodynia were rare and nonexistent, respectively.

For diseases of the lungs, segmental signs were very widespread and covered a range from V2 to L2. Signs were generally less focused than for the heart, and no clear maximum was discernible. In this, the distribution deviated from Hansen and Schliack’s [33], who reported T9 as the lower margin of segmental signs in lung diseases. As was to be expected due to the mixture of left, right, and bilateral organ diseases, no lateralization could be seen.

For the stomach, the segmental distribution was almost strictly thoracic, from T2 to T12, with a maximum at T6-9 on the front and on the back. Similar to the heart, superficial hyperalgesia for the stomach was lateralized to the left, as predicted by the side rule. The comparison with Hansen and Schliack [33] showed that the maximum of signs in T6-9 fell in the expected range in the front view. On the back, however, there was only a partial overlap, with Hansen and Schliack [33] predicting higher thoracic segments than were found in our study.

The similarity with Hansen and Schliack’s [33] results was much higher for patients with liver/gallbladder diseases. Here, segmental signs showed a largely thoracic distribution but with the characteristic shoulder presentation in segments C3-C5 [33]. In terms of lateralization, muscle resistance, defense, and superficial and deep hyperalgesia were predominantly right-lateralized, with superficial hyperalgesia (Head zone) showing almost complete right-lateralization.

Finally, segmental signs of the kidneys had the narrowest distribution, starting at T6 and extending down to L2, once again, showing a rather high similarity with the predicted distribution by Hansen and Schliack [33].

### Comparison of Spontaneous Pain and Segmental Signs

**Figure 5:**
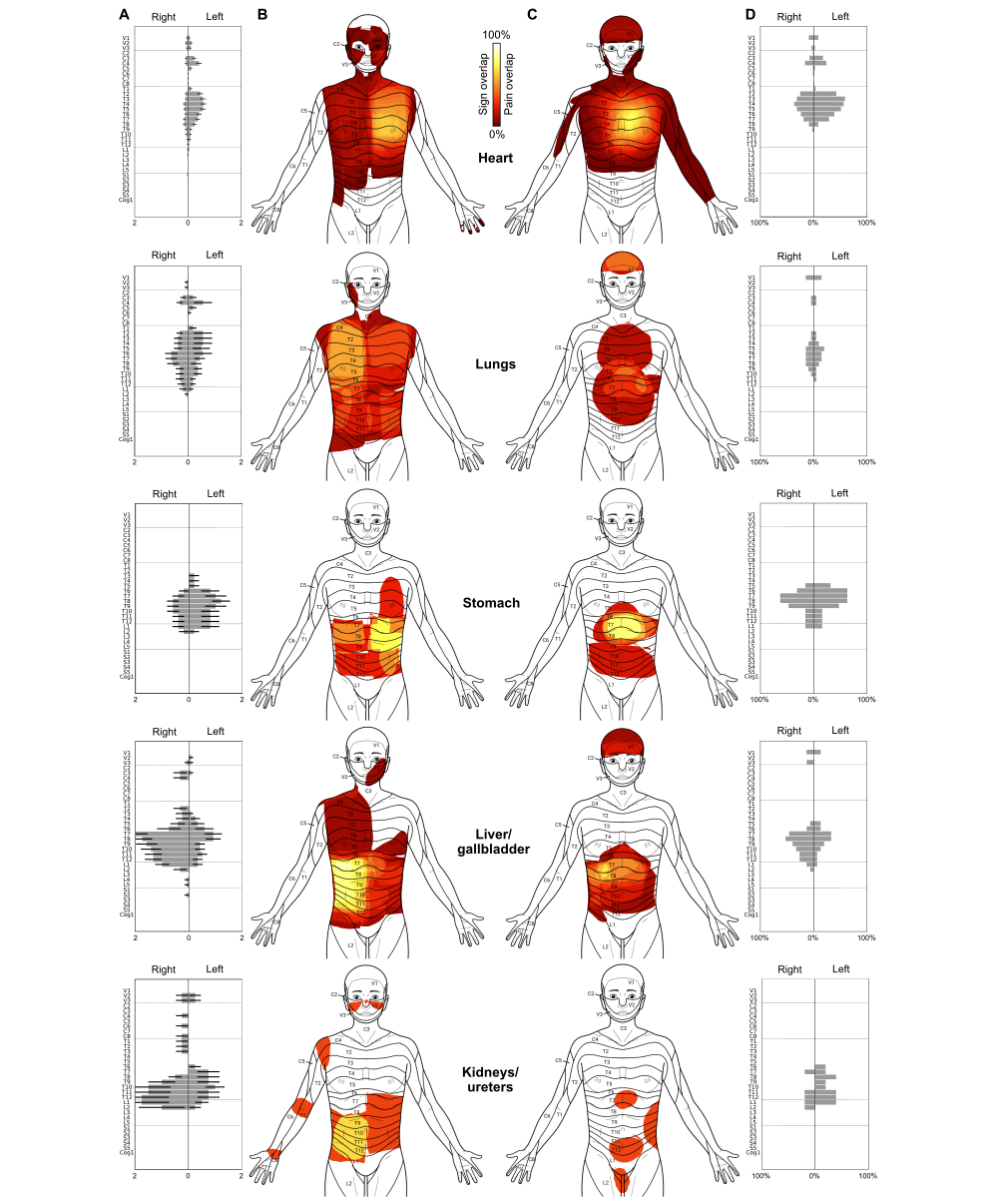
Direct comparison between segmental signs (A and B) and spontaneous pain (C and D) in acute visceral diseases. Column A shows the mean number of segmental signs per segment for the individual organs, and column B shows the joint distribution of segmental signs (cf Figure 2). The symptom of spontaneous pain is shown as mean distributions of pain in column C, and their exact segmental content is shown in column D.

The segmental distributions of spontaneous pain and segmental signs are shown in Figure 5. It is evident that spontaneous pain differed markedly from segmental signs. It spanned fewer segments but extended to the head region (V1) in cardiac, respiratory, and liver/gallbladder affections. Furthermore, spontaneous pain was much less lateralized than segmental signs and, instead, rather localized in the body midline. In general, the pain was less widespread and showed much weaker lateralization than segmental signs in the unpaired organs (ie, heart, stomach, and liver/gallbladder).

## Discussion

### Principal Findings

In this study, we investigated bodily patterns and lateralization of segmental signs and spontaneous pain in acute visceral diseases. We derived mean distributions of spontaneous pain and segmental signs for the heart, lungs, stomach, liver/gallbladder, and kidneys/ureters by combining digital pain drawing technology and a structured 10-minute bodily examination in patients presenting to the emergency room. We extracted precise information on the segmental content and lateralization and compared the results with the slightly outdated but authoritative German work of Hansen and Schliack [33]. Although purely descriptive by design, our study is the first in the English language to provide a detailed account of simultaneously collected segmental signs and symptoms for visceral diseases in the clinical setting.

### Lateralization of Segmental Signs

The lateralization of segmental signs is important, as it allows one to quickly identify the affected body side (ie, the side hosting the affected organ; Table 1). This side rule may be useful in the differential diagnosis, such as in differentiating gastritis from hepatitis or pancreatitis, or acute coronary syndrome from a pulmonary embolism or esophagitis. Due to our study design and the very mixed patient sample, our results regarding lateralization were limited to the heart, stomach, and liver/gallbladder. Although segmental signs of the lungs and kidneys/ureters are also expected to be found ipsilateral to the affected side, a separate analysis for the individual sides was not possible for these organs due to the limited number of cases, many of which showed bilateral affections.

Although our data were not analyzed prospectively, it appears, for the heart, stomach, and liver/gallbladder, that they may support the findings of Hansen and Schliack [33] that segmental signs appear ipsilateral to the affected organ. While this was evident for the mean bodily distributions of segmental signs, we also found an ipsilateral occurrence of mydriasis, a finding rarely raised outside the neurological setting. It results from a reflex mediated by the ciliospinal center, which conducts impulses from the entire body to the sympathetically innervated dilator pupillae muscle (p271) [28] and, more than 100 years ago, was first described to occur in affections of the lungs [36] and the heart [37]. More recently, Rosenberg [38] has shown that anisocoria (ie, unequal pupil size) under physiological conditions is a manifestation of sympathetic asymmetry.

We found ipsilateral (ie, right-sided) mydriasis in 42% of our liver/gallbladder patients and not a single case of contralateral mydriasis. For the heart, mydriasis was less frequent (24% ipsilateral vs 5% contralateral), yet this means that patients showing the sign had it on the ipsilateral side in almost 83% of the cases. Hansen and Schliack [33] reported qualitatively similar but generally higher numbers for mydriasis. In their sample of 28 heart patients, 27 (96%) had mydriasis, and this was ipsilateral in 26 patients (96%). In 56 liver/gallbladder patients, 54 (96%) had mydriasis, of which 50 cases (93%) were ipsilateral (ie, right-sided).

The generally higher numbers of mydriasis in heart diseases found in Hansen and Schliack’s [33] work may be explained by the fact that these authors used dark adaptation and infrared photographs in many of their patients, which made their examination less subjective, while our examiners were restricted to visual inspection under normal light. Clinicians interested in this phenomenon should consider using a portable infrared pupilometer.

### Localization and Distribution of Segmental Signs

A subset of the findings collected in our study was further analyzed to extract detailed segmental information. We called this group of findings “distributed signs.” It comprised a number of somatosensory (ie, superficial and deep hyperalgesia and allodynia), somatomotor (ie, superficial skin resistance, muscle resistance, and defense), and visceromotor signs (ie, vasomotor changes, piloerection, and asymmetric hyperhidrosis). Of these, superficial hyperalgesia (ie, Head zones), muscle resistance, defense, and deep hyperalgesia (ie, Mackenzie zones) were the most frequently observed in our sample of patients, while others, such as allodynia, piloerection, asymmetric hyperhidrosis, or zoster, were exceedingly rare.

There was a close similarity between the original maps of segmental signs by Hansen and Schliack [33] and our mean distributions of all signs (Figure 3). For a prospective evaluation, however, future studies should aim to quantify this similarity (eg, by using spatial similarity measures).

Several groups have studied individual segmental signs or groups of signs since the days of Hansen and Schliack. For example, Nicholas and colleagues [39] found that patients with myocardial infarction showed characteristic paravertebral soft tissue changes readily detected by palpation. Compared with patients without diagnosed cardiovascular diseases, patients with myocardial infarction had a significantly higher incidence of increased firmness, warmth, ropiness, oedematous changes, and heavy musculature, almost entirely confined to cardiac segments T1-4. In a follow-up 3 years after the infarction, these signs had regressed in the majority of patients [40]. Vecchiet and colleagues [41] found ipsilateral superficial and deep hyperalgesia of the first lumbar (L1) segment in patients after renal/ureteral calculosis.

For the gallbladder, Stawowy and colleagues [42] found that all patients with acute cholecystitis reported referred pain in the epigastrium and under the right curvature. Segmental signs inside this area were quantitatively evaluated using von Frey hairs, warm and cold metal rollers, and a constant current stimulator to test for the different forms of hypersensitivity or allodynia. The authors reported that 20% of the patients showed hypersensitivity or allodynia to mechanical, 53% to cold, 40% to warmth, and 63% to electrical stimulation [42]. The same authors reported that 50% to 56% of patients with acute appendicitis showed segmental signs over the right abdominal quadrant, with the maximum located approximately at McBurney point [43]. These findings were recently confirmed by Roumen and colleagues [44], who reported that 39% of patients with acute appendicitis demonstrated at least one segmental sign (ie, hyperalgesia, hypoesthesia, altered cool perception, or positive pinch test) over the lower right abdomen. Finally, a large number of smaller studies and case reports have been published, which have been reviewed by Beal [45].

### Segmental Signs Versus Spontaneous Pain

The majority of our patients with visceral diseases reported spontaneous pain (Table 3). In 85% of the cases, it was, by far, the most frequent finding, followed by superficial hyperalgesia (46%), nausea (45%), and muscle resistance (39%).

Many textbooks assign pain location a discriminative role in the differential diagnosis (eg, retrosternal chest pain that radiates to the left arm or lower jaw usually refers to acute coronary heart disease. However, such predictive power of pain location has been a matter of debate for decades [11-13]. Here, we found, by direct comparison of spontaneous pain and segmental signs, that the two were rather dissimilar in their bodily patterns and segmental distributions (Figure 5). Irrespective of the affected organ, spontaneous pain was less widespread than segmental signs (ie, it included fewer segments). Furthermore, spontaneous pain appeared mostly in the body midline, thus lacking the diagnostically relevant ipsilateral distribution seen in the majority of segmental signs. As Figure 5 shows, patients with lung, stomach, and liver/gallbladder diseases all showed spontaneous pain in the epigastric region (T5-9), thus rendering this symptom unsuitable for differential diagnosis.

The substantial differences found between pain and segmental signs regarding their location and lateralization underline the importance of making a clear distinction between visceral pain, (referred) hyperalgesia, and other segmental signs.

Despite the purely descriptive design of this study, our results (Figures 4 and 5) regarding the benefit of using spontaneous pain or segmental signs seem to favor the latter over the former. Future studies should test this in a prospective way (eg, by letting a blinded assessors predict the affected organ from the distribution of spontaneous pain or from that of segmental signs).

### Limitations

Our study had several limitations that need to be discussed. Firstly, our patient sample was relatively small, as we could only analyze approximately half of the included patients. There were two reasons for this. On the one hand, approximately one-quarter of our patients had to be excluded from the analysis, since they left the hospital without a confirmed final diagnosis. This is due to the unique situation in the emergency room, where the vital role of the specialist is to rule out life-threatening conditions. A further one-quarter of the remaining patients had to be excluded from the analysis because they suffered from diseases affecting multiple organs. Secondly, while we took great care to include only patients with single-organ problems, it is likely that affections of other organs were present but overlooked in some of the patients. This means that some patients who seemed to only present with cardiac disease may have had another underlying disease affecting other organs. Thirdly, findings collected by means of palpation are naturally more subjective than, for example, laboratory results. While there are ways to measure segmental signs more quantitatively, we did not do so to keep the examination time to an absolute minimum, as required by the clinical setting. Finally, we did not differentiate explicitly between signs and symptoms that patients had only during their acute problem from patient symptoms that occurred usually. This may have introduced some bias.

### Conclusions

This study underlines the usefulness of including segmental signs in the bodily examination of patients with acute medical problems. As we have shown, capturing the location of segmental signs on a digital body map may assist in the clinical decision-making process in some acute visceral conditions. Segmental information and lateralization from the 3 most-frequent signs (superficial hyperalgesia, muscle resistance, and mydriasis) can be quickly acquired and may help physicians narrow the differential diagnosis.

## Appendix

Multimedia Appendix 1 Supplementary table 1.

Multimedia Appendix 2 Supplementary figure 1.

Multimedia Appendix 3 Supplementary table 2.
