# Supplementary material for: Lateralization and Bodily Patterns of Segmental Signs and Spontaneous Pain in Acute Visceral Disease: Observational Study
Source: J Med Internet Res. 2021 Aug 27;23(8):e27247. doi: 10.2196/27247 (PMC8459716; doi:10.2196/27247)
Supplement: Multimedia Appendix 1 [file jmir_v23i8e27247_app1.pdf]

| Affected organ | Diagnosis | ICD-10-CM | n | % |
|---|---|---|---|---|
| **Heart** | Unstable Angina | I20.0 | 29 | 46.0 |
| | Non-STEMI | I21.4 | 8 | 12.7 |
| | Atrial Fibrillation | I48.0 | 14 | 22.2 |
| | LV Decompensation | I50.1 | 5 | 7.9 |
| | Valvular disease | I08.0 | 2 | 3.2 |
| | Endocarditis | I33.0 | 1 | 1.6 |
| | STEMI | I21.1 | 2 | 3.2 |
| | ASD | Q21.1 | 1 | 1.6 |
| | Palpitations | R00.2 | 1 | 1.6 |
| | Total | | 63 | |
| **Liver/ Glallbladder** | Hepatitis | B17.9 | 3 | 25.0 |
| | Intrahepatic bile duct carcinoma | C22.1 | 1 | 8.3 |
| | Liver metastasis | C73 | 2 | 16.7 |
| | Calculus of gallbladder with acute cholecystitis | K80.2 | 4 | 33.3 |
| | Obstruction of bile duct | K83.1 | 1 | 8.3 |
| | Cystic disease of liver | Q44.6 | 1 | 8.3 |
| | Total | | 12 | |
| **Lung** | Tuberculosis | A15 | 1 | 6.3 |
| | Adenocarcinoma | C34 | 1 | 6.3 |
| | Pneumonia or Bronchitis | J15 | 7 | 43.8 |
| | COPD exacerbation | J44.1 | 3 | 18.8 |
| | Asthma exacerbation | J45.51 | 1 | 6.3 |
| | Bronchial cyst rupture | J98.4 | 1 | 6.3 |
| | Interstitial lung disease | M34.82 | 1 | 6.3 |
| | Complications of lung transplant | T86.81 | 1 | 6.3 |
| | Total | | 16 | |
| **Stomach** | Acute gastritis | K29.0 | 4 | 80.0 |
| | Gastro-esophageal reflux disease without esophagitis | K21.9 | 1 | 20.0 |
| | Total | | 5 | |
| **Kidneys/ Ureter** | Acute pyelonephritis | N10 | 1 | 25.0 |
| | Unspecified renal colic | N23 | 2 | 50.0 |
| | Urinary tract infection, site not specified | N39.0 | 1 | 25.0 |
| | Total | | 4 | |
