# Supplementary material for: Lateralization and Bodily Patterns of Segmental Signs and Spontaneous Pain in Acute Visceral Disease: Observational Study
Source: J Med Internet Res. 2021 Aug 27;23(8):e27247. doi: 10.2196/27247 (PMC8459716; doi:10.2196/27247)
Supplement: Multimedia Appendix 2 [file jmir_v23i8e27247_app2.pdf]

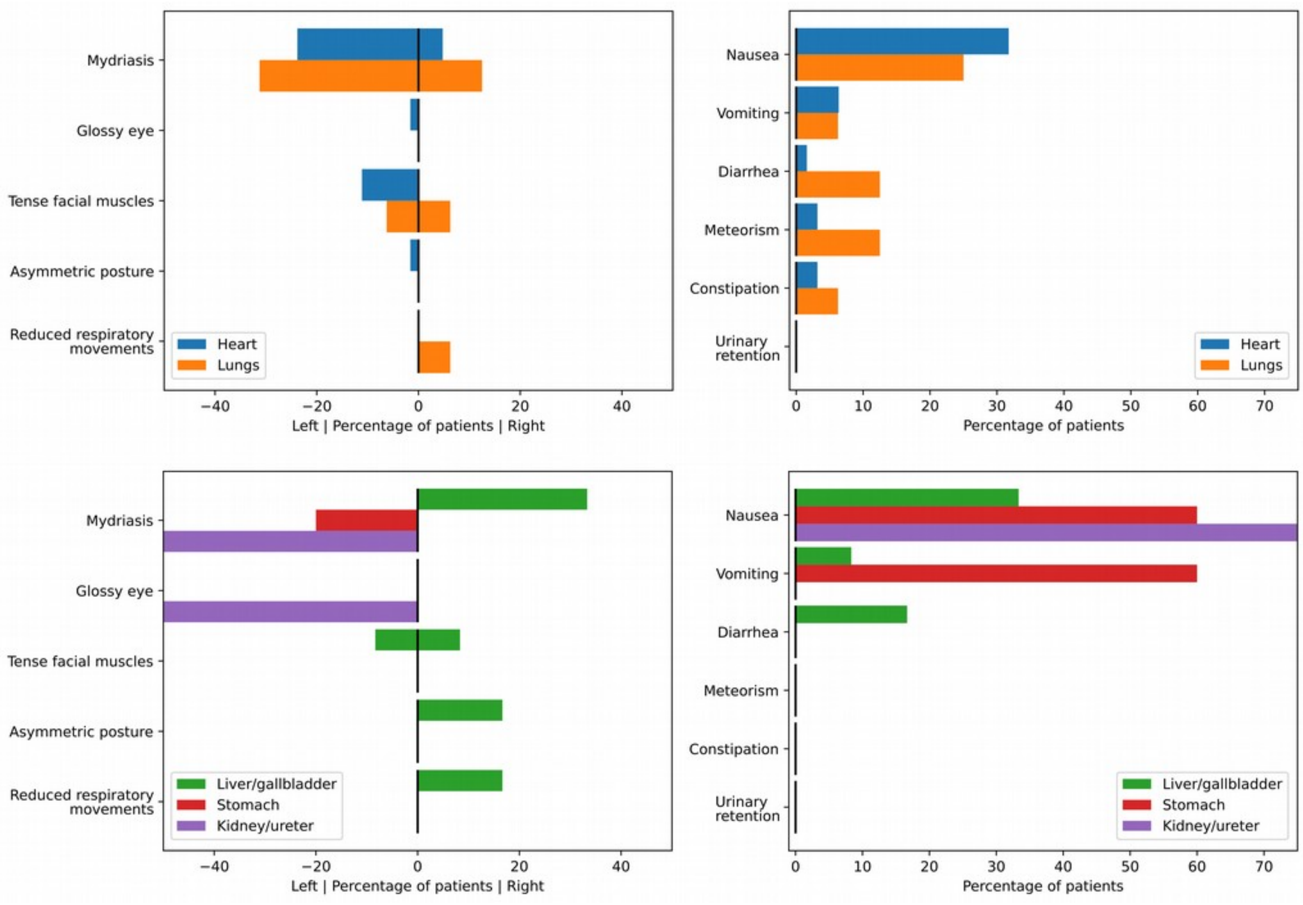

Supplementary Figure 1. Lateralization signs (left) and segmental symptoms (right) for chest organs (top) and abdominal organs (bottom).
