# Supplementary material for: Lateralization and Bodily Patterns of Segmental Signs and Spontaneous Pain in Acute Visceral Disease: Observational Study
Source: J Med Internet Res. 2021 Aug 27;23(8):e27247. doi: 10.2196/27247 (PMC8459716; doi:10.2196/27247)
Supplement: Multimedia Appendix 3 [file jmir_v23i8e27247_app3.pdf]

Supplementary Table 1. Demographics and primary diagnoses for the individual patients with single-organ affections shown in Figure 2.

Organ	Patient	Reason to seek medical help	Age	Sex	Primary diagnosis
Heart	**1**	Chest pain	72	W	Unstable angina
	**2**	Chest pain	80	W	Unstable angina
	**3**	Chest pain	27	M	Rheumatic disorders of both mitral and aortic valves
	**4**	Chest pain	66	M	Unstable angina
Lungs	**5**	Dyspnea with cough	64	W	Other disorders of lung
	**6**	Dyspnea	77	W	Chronic obstructive pulmonary disease with (acute) exacerbation
	**7**	Dyspnea	42	W	Acute bronchitis
	**8**	Chest pain	76	M	Chronic obstructive pulmonary disease with (acute) exacerbation
Stomach	**9**	Abdominal pain	60	W	Acute gastritis
	**10**	Abdominal pain	48	W	Unspecified chronic gastritis
	**11**	Abdominal pain	33	M	Acute gastritis
	**12**	Abdominal pain	46	W	Gastro-esophageal reflux disease without esophagitis
Liver/ Gallbladder	**13**	Abdominal pain	30	W	Calculus of gallbladder without cholecystitis
	**14**	Chronic diarrhea	66	W	Malignant neoplasm of thyroid gland
	**15**	Elevated liver values	53	M	Inflammatory liver disease, unspecified
	**16**	Abdominal pain	43	W	Calculus of gallbladder with acute cholecystitis
Kidneys/ Ureters	**17**	Fever	26	M	Urinary tract infection with unspecified site
	**18**	Abdominal pain	39	M	Unspecified renal colic
	**19**	Abdominal pain	63	M	Unspecified renal colic
